# Intestinal occlusion through extrinsec stenosis of transverse colon associated with internal right mesocolic hernia

**Published:** 2012-09-25

**Authors:** R Balanescu, L Topor, S Tala, A Ulici, B Frumuseanu

**Affiliations:** Pediatric Surgery Department, “Grigore Alexandrescu” Children Clinical Emergency Hospital, Bucharest

**Keywords:** malrotation, intestinal obstruction, transverse colon stenosis, internal right mesocolic hernia

## Abstract

**Rationale.** The incidence of malrotation has been estimated at 1 in 600 live births. An increased incidence of 0,2% has been found in barium swallow studies, whereas autopsy studies estimate that the true incidence may be high as 1% of the total population. The clinical manifestations are elusive; therefore, the diagnosis must be based on the presence or absence of the acute obstruction. Radiologic investigations, especially those using contrast substances, are the ones used most often in the diagnosis of malrotation. Laparoscopy may give the clinician a valuable tool that will help him diagnose the rotational anomalies and correct the potentially obstructing lesions with minimal surgical trauma to the patient. The role of the surgical treatment is to prevent volvulus and to treat any kind of obstruction. Ladd’s procedure provides all the elements for reaching this goal.

**Objective.** The following report describes a particular case of one female patient, 8 years old, admitted in our clinic with signs of intestinal obstruction. She had similar episodes in the last three months, but the symptoms had resolved spontaneously.

**Methods and results.** Upper gastrointestinal series showed an anomaly of rotation and barium enema discovered a tight stenosis on the transverse colon. Emergency surgery using laparotomy enabled diagnosis. Intraoperatively, a right mesocolic hernia and a transverse colon extrinsic stenosis due to abnormal peritoneal attachments were noted.

**Discussion. **Colon obstruction due to peritoneal bands is extremely rare. The clinical manifestations are not specific and we need radiologic procedures to help diagnose the disease. The cause of the obstruction is not always evident despite the availability of modern imaging techniques. Since preoperative diagnosis is difficult, morbidity and mortality can be decreased by an early surgical intervention.

## Introduction

Malrotation is a congenital abnormal position of the bowel within the peritoneal cavity and it usually involves both the small and the large bowel. Malrotation is accompanied by abnormal bowel fixation by mesenteric bands or absence of fixation of portions of the bowel, leading to increased risks of bowel obstruction [**1**].

Midgut malrotation has been estimated to occur in approximately one in 5000 live births. However, it is difficult to ascertain the true incidence because this condition will go undetected during childhood [**2**]. The vast majority of the complications associated with midgut malrotation present in the first month of life and 60-85% of the cases are diagnosed in this age group [**3**]. However, malrotation is a rare cause of intestinal obstruction – only 2% of the cases.

Clinical manifestations are not specific. It is important to determine if the disease is acute or chronic and to determine the eventual presence of midgut volvulus. Due to Ladd’s bands or intermittent midgut volvulus, the acute bowel obstruction can present with vomiting, can be typically bilious, as the commonest presenting feature accompanied by colicky abdominal pain and abdominal distention. Usually, it manifests itself in the first year of life. Older children without acute volvulus more often present with chronic episodic obstructive symptoms, failure to thrive, malabsorption, diarrhoea and non-specific colicky abdominal pain [**4**]. Lack of fixation of the mesentery of the right or left colon, or of the duodenum, may result in the formation of potential spaces for hernias. Internal hernias are associated with recurrent entrapment of bowel with partial obstruction, which may eventually progress to complete obstruction [**5**]. Anatomically, six different types of intra-abdominal hernias are distinguished: The left- and right-sided paraduodenal hernias and the transverse mesenteric hernias represent the most frequent form of internal hernia in children [**6**]. Signs and symptoms of an internal hernia are related to recurrent, intermittent bouts of intestinal obstruction characterized by recurrent colic [**5**].

The diagnostic is difficult because of a lack of specificity of the clinical manifestation, the malrotation being rarely considered a cause of intestinal obstruction. Up to 10% of the diagnoses of malrotation are made as an incidental finding [**4**]. Apart from clinical symptoms, radiological procedures are most useful. A plain X-ray film of the abdomen and the use of intestinal contrast medium may show displaced small intestinal loops or signs of intestinal obstruction. CT and ultrasonography can be used as well in the preoperative diagnostic [**6**].

The surgical intervention must be an urgent procedure. The purpose of treatment of rotational anomalies is to prevent midgut volvulus and relieve any obstructions. Children with rotational anomalies without volvulus can be initially evaluated laparoscopically. Laparoscopy provides the surgeon a valuable tool that helps him diagnose rotational anomalies and the correct potentially obstructing lesions with minimal surgical trauma to the patient. If the midgut volvulus is found, the difficulty of laparoscopic procedure is highly increased, being recommended to the most experienced laparoscopists. In this case, we recommend an open surgical procedure [**7**].

## Methods 

An 8-year-old girl was admitted to the hospital in emergency conditions due to vomiting, colic abdominal pain, constipation and abdominal distension. Three months previously, she presented a similar episode, but the symptomatology disappeared

## Results 

The upper gastrointestinal series showed an abnormal position of the duodenojejunal junction and the first jejunal loops in the right superior quadrant.

**Fig. 1:**
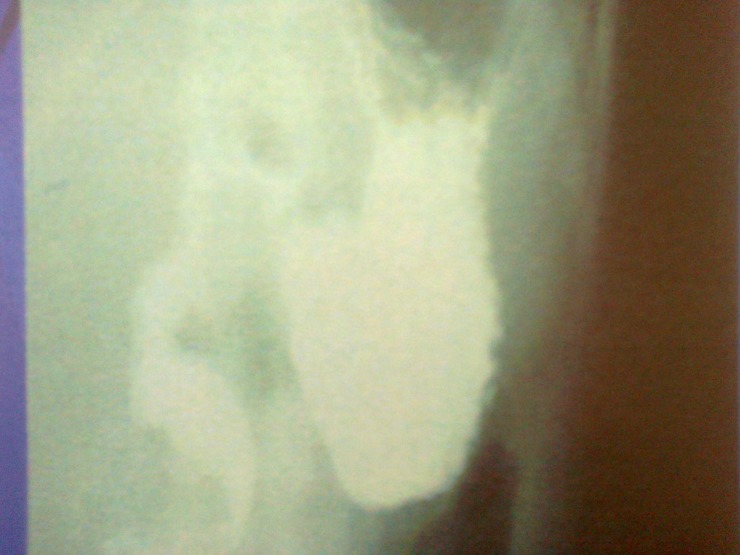
Upper gastrointestinal series

Barium enema demonstrated a normal position of the rectum, sigmoid and descendent colon, but on the middle part of the transverse colon, it showed a tight stenosis, of 3-4 cm length, then the passage of the contrast substance stopped. 

**Fig. 2:**
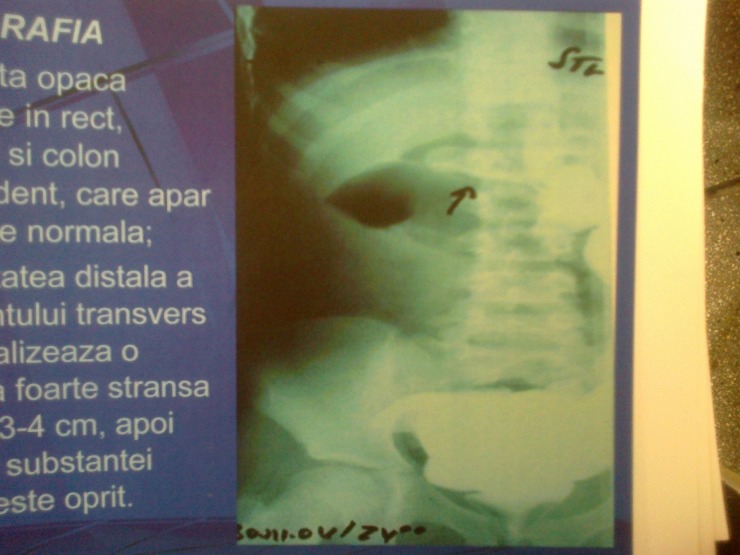
Barium enema with transverse colon stenosis

Taking in consideration the clinical manifestations and the results of radiologic investigations we established a preoperative diagnostic of intestinal obstruction and chose to operate immediately; the final diagnostic being an intraoperatory finding. Intraoperatory, we discovered a rotational anomaly with cecum situated on the right superior quadrant. In the middle of the transverse colon, there were abnormal peritoneal coloparietal bands, which were the cause of a tight stenosis.

**Fig. 3:**
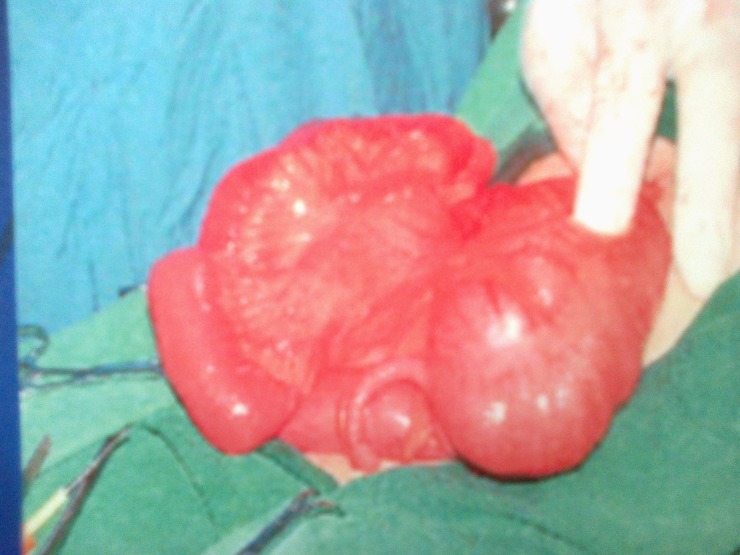
Transverse colon stenosis

After sectioning the bands and the mobilization of the cecum and transverse colon to the left, we discovered a right mesocolic hernia.

**Fig. 4:**
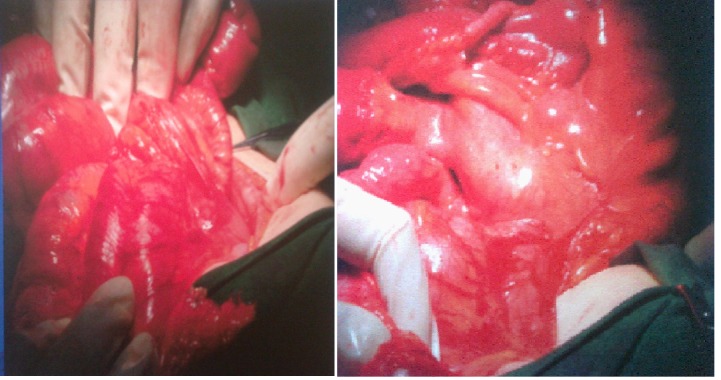
Internal right mesocolic hernia

Inside of it, there were the first jejunal loops. We chose the Ladd procedure with the mobilization of the intestinal loops on the right and the colon on the left. The final diagnostic of intestinal obstruction through extrinsic stenosis of transverse colon associated with internal right mesocolic hernia was established intraoperatory.

## Discussion

Malrotation is an unusual cause of intestinal obstruction; it represents 2% of cases. Colon obstructions due to peritoneal bands are extremely rare. There is just one case cited in the reviewed cases [**8**]. In our case, the intestinal obstruction was caused by a very tight stenosis of the transverse colon made by peritoneal bands. A high percent of the intestinal obstruction cases, same as our case, manifest themselves with recurrent abdominal pain, vomiting and constipation. The older children had a longer course of vague, antecedent symptoms such as the one described previously [**9**]. The pre-operatively diagnostic of intestinal obstruction was made with the help of radiologic procedures. In the same time, we established the diagnostic of malrotation. In a study made by Powell on 70 children with malrotation, the upper gastrointestinal (GI) series revealed the diagnosis in 41% cases, as did contrast enema in 34% [**9**]. The cause of obstruction may be difficult to establish pre-operatively despite the availability of modern imaging techniques [**10**]. The final diagnostic in our case was made intraoperatory because the internal hernia is a diagnostic difficult to make preoperatively [**11**]. CT is believed to facilitate the diagnosis of internal abdominal herniations. The use of CT could limit the rate of misdiagnosed internal abdominal herniations, because subtle transmesenteric internal abdominal herniations can be difficult to diagnose on laparoscopy [**12**]. The differential diagnostic of the patients with recurrent abdominal pain must always take into consideration malrotation. The awareness of this diagnostic may help reduce delays in diagnosis and surgical treatment [**13**]. A high index of suspicion in children with bilious vomiting, especially when recurrent, undelayed diagnosis and prompt surgical intervention may give a favorable prognosis in children with malrotation [**14**]. Laparoscopy has a role in diagnostic as well as treatment of the malrotation. In our case, due to emergency character of the disease – intestinal obstruction with undiagnosed cause – we chose open surgery. The surgical treatment must be prompt in order to prevent the devastating complications. The Ladd procedure is the treatment of choice in this case, and we did it as well in our case. The mortality and morbidity associated with malrotation can be lowered only with the help of surgical treatment.
